# Meanings attributed to the voice by transgender women in socialization processes

**DOI:** 10.1590/0034-7167-2025-0168

**Published:** 2026-06-12

**Authors:** Tiago José Nunes de Aguiar, Christina César Praça Brasil, Jonas Loiola Gonçalves, Francisca Francisete de Sousa Nunes Queiroz, Waléria Tomaz Pacífico, Juliana Carneiro Melo

**Affiliations:** I Universidade de Fortaleza. Fortaleza, Ceará, Brazil; II Universidade Estadual do Ceará. Fortaleza, Ceará, Brazil

**Keywords:** Transgender Women, Gender Identity, Voice, Speech Therapy, Social Inclusion., Mujeres Transgénero, Identidad de Género, Voz, Terapia del Habla, Inclusión Social.

## Abstract

**Objectives:**

to understand the meanings attributed to voice by transgender women in the socialization process.

**Methods:**

qualitative research, including 13 transgender women. The study was conducted with focus groups. Data were analyzed using thematic content analysis, interpreting them in light of Symbolic Interactionism.

**Results:**

among the emerging topic for this study, the following was selected: “Meanings attributed to voice and insecurities in oral communication from transgender women’s perspective in socialization processes”, demonstrating that the meanings attributed to voice permeate feelings of fear, shame, and acceptance (self and social).

**Conclusions:**

for transgender women, voice is an essential resource for their inclusion and personal and social acceptance, requiring attention from the healthcare system to promote comprehensive care and reduce the vulnerability of this population. Qualitative studies highlight subjective nuances that can aid in the implementation of interdisciplinary healthcare services.

## RESUMEN

**Objetivos:**

comprender los significados que las mujeres transgénero atribuyen a la voz en su proceso de socialización.

**Métodos:**

investigación cualitativa con 13 mujeres transgénero. El estudio se realizó mediante grupos focales. Los datos se analizaron mediante análisis de contenido temático, interpretándolos a la luz del Interaccionismo Simbólico.

**Resultados:**

entre los temas emergentes de este estudio, se seleccionó el siguiente: “Significados atribuidos a la voz e inseguridades en la comunicación oral desde la perspectiva de las mujeres transgénero en el proceso de socialización”, lo que demuestra que los significados atribuidos a la voz permean sentimientos de miedo, vergüenza y aceptación (propia y social).

**Conclusiones:**

para las mujeres transgénero, la voz es un recurso esencial para su inclusión y aceptación personal y social, lo que requiere la atención del sistema de salud para promover la atención integral y reducir la vulnerabilidad de esta población. Los estudios cualitativos resaltan matices subjetivos que pueden ayudar en la implementación de servicios de salud interdisciplinarios.

## INTRODUCTION

Gender inequalities are understood as a phenomenon involving socio-historical and cultural processes, with unequal power relations often tied to hierarchies between being “man” and “woman”. These processes can act as artifacts for social exclusions, “normality”, and repeated (dis)affective pedagogies attributed to women^(1)^.

Transgender people are those who do not identify with their biological sex as declared at birth by others, and may therefore suffer repeated social exclusions related to their identity. In search of gender identity, the transgender population (or trans population) seeks healthcare services to transition, seeking their social construction and sense of belonging. This can often be motivated by the oppression of the heteronormativity movement embedded in a sexist and sexist society^(2)^.

In the Brazilian scenario, even with the existence of the Brazilian Healthcare System (In Portuguese, *Sistema Único de Saúde* - SUS), many trans women still suffer from difficulties in accessing healthcare services, mainly due to the almost non-existent information about a population that is still stigmatized and vulnerable^(2-5)^. In recent years, the demand for specialized public healthcare services by transgender women (or trans women) has been increasing, especially for those seeking sex reassignment surgery or transgenitalization (surgery to adapt anatomical sex to the gender with which a person identifies). Furthermore, the lack of structured care for the trans population and the prejudice of professionals in healthcare services are perceived as barriers to recognizing that the population should be served at all levels of healthcare. This highlights the fragile provision of care in both international and national experiences^(3-8)^.

It is important to highlight that training the interdisciplinary team is a fundamental aspect in the transsexualization process, as it involves a broad transformation of the body, behavior, social and philosophical concepts, and image equivalent to gender identity; and these changes require that individuals be informed and supported in this journey^(9,10)^. To this end, physicians, psychologists, speech therapists, and other professionals make important contributions in this context, since adjustments to physical and mental health are a reality and permeate all stages from the moment a person assumes their gender identity. Thus, the voice, as an element of expressiveness, needs to be cared for and observed, providing trans people with greater comfort and quality of life in terms of vocal health and social relationships^(1,5,11)^.

The voice is the main means of communication for human beings, since when associated with speech it carries prosodic elements, segmental variants, rhythmic patterns, and vocal quality adjustments, forming a person’s vocal identity^(9-11)^. In the context of trans women, voice plays a fundamental role in the construction of identity. Therefore, it has been highlighted as one of the main complaints of this group, since the listener’s identification of a male voice in a trans woman brings them insecurity, fear, shame, and social embarrassment. There are even numerous reports in literature and the media in general of prejudice, physical, and verbal aggression against this group^(12)^.

Therefore, many of them seek speech therapy and surgery to identify as female in everyday situations, especially in cases where the listener lacks visual cues. To disguise their voices in social settings, trans women end up making adjustments to their vocal tracts to characterize a feminine voice. However, this “new voice” often falls far short of the vocal standards expressed in the heteronormativity imposed by society^(8,9)^.

This inadequacy in trans women’s voices is caused by a lack of professional guidance, which leads to extreme vocal fatigue at the end of the day and a lack of identification with their voice. They also report that these vocal adjustments are impossible to maintain for a long period and end up being noticed by listeners. Furthermore, trans women experience daily judgments of their voices by listeners, which can have a significant impact on their subjectivities and pluralities as they construct their new gender identity^(12,13)^.

In Brazil, there is a need to produce knowledge about the perception of trans women’s voices and the socialization process, given the lack of consolidated research from an interactionist perspective. Therefore, the construction of this evidence is urgent given the calls to overcome gaps and improve healthcare. Knowledge contributes to the development of comprehensive care and to achieving the Sustainable Development Goals, especially goal 3, which addresses ensuring and promoting healthy lives for all.

Therefore, it is imperative to broaden the comprehensive view of transgender women’s health, favoring the socialization of this population, and the question is: what meanings are attributed to the voice by trans women in the socialization processes?

## OBJECTIVES

To understand the meanings attributed to the voice by transgender women in socialization processes.

## METHODS

### Ethical aspects

This research was approved by the *Universidade de Fortaleza* Research Ethics Committee. It is important to note that, to protect participants’ identities, the coding adopted was letters, numbers, age, and occupation, referring to, for example, “TW1, age and occupation”, and so on. It is also important to note that all participants were invited and agreed to participate in the study by reading and signing the Informed Consent Form.

### Study design

This is a qualitative study^(14)^ guided by the COnsolidated criteria for REporting Qualitative research^(15)^. The approach used permeates the subjectivity of trans women and their socio-historical processes. It is pointed out that, within the scope of this investigation, the analysis of the following aspects is fundamental: participants’ culture, feelings, beliefs, perceptions, habits, knowledge, life experiences, among others^(14)^.

Thus, the typology introduces nuances into knowledge production for understanding the meanings of voice for trans women in socialization processes, given that, due to the lack of care in healthcare services, as well as oppressive practices in society, these women are often objectified and neglected. Thus, qualitative research introduces a perspective for qualified listening to this population, based on respect for their social processes and their historical background^(12,14)^.

### Study setting and participant selection

The study was conducted from August to December 2023. Initially, two referral centers for transgender care were identified for the study, both located in Fortaleza, Ceará, Brazil. One of the centers was a private clinic staffed by otorhinolaryngology and speech-language pathology professionals who serve patients with health insurance and private plans.

It should be noted that the trans patients treated at this service are residents of Fortaleza, Ceará, other Brazilian states, and other countries. Another research site was a healthcare service affiliated with a private university, where services are provided to the trans population through the SUS and through health insurance plans, with specialized outpatient clinics in the areas of psychology, speech therapy, otolaryngology, and gynecology.

In the second stage, participants were selected using the snowball method, a recruitment strategy based on the use of referral chains. This technique is useful for studying groups with limited or difficult access, enabling the recruitment of the target audience and, based on eligibility criteria, entry into the study^(14)^. The search for trans women began at the two healthcare services mentioned above, where posters were displayed and invitations were posted on social media (Instagram^®^). This process enabled the acquisition of key informants, who recommended other participants for the study and approached them about their participation using these strategies.

The inclusion criteria focused on: individuals who identify as transgender or transsexual women aged 18 to 60 (as adulthood excludes aspects of vocal change and aging due to age); Brazilian trans women, residing in any Brazilian city or in other countries; and who had a connection with the services under investigation or with women treated at these services. Thus, 13 trans women were identified as eligible for inclusion in the study.

### Data production

Data production occurred in two stages. Initially, a socioeconomic and health questionnaire was administered to trans women, developed by the researchers themselves. This questionnaire collected data on age, health conditions, age of gender transition, occupation, hormonal therapy, access to speech-language pathology and otolaryngology, current vocal conditions, and other aspects.

Consequently, a focus group was developed, which covered guiding questions with topics developed from the premises of Symbolic Interactionism (meanings, actions and interpretations), addressing the following aspects: perceptions and feelings about gender identification through the voice; associations between quality of life and voice; interference of the voice in social and work relationships; pain and anguish that associate the voice with the transsexualization process; psychosocial impacts perceived by the vocal condition; trans women’s hopes and needs related to general and vocal health.

It is noteworthy that both focus groups were conducted virtually, led by two speech-language pathologists (one male and one female), both with prior experience in qualitative research and trained with the study instrument. Dates and times were agreed upon between the participants and the researchers. A Google Meet^*®*^ account with a Meet Pro license was used, and a link to the session was provided. The produced material was later downloaded to the researchers’ personal files and supplemented with field journal notes.

### Data organization and analysis

The data were transcribed based on the recordings and notes, aiming for greater reliability, richness of detail, and necessary additions to the records. Subsequently, they were treated based on thematic content analysis^(14)^, which corresponds to a method that encompasses a set of strategies that enable the analysis of various types of communication, with the objective of obtaining greater knowledge about the subject at hand within a more specific context.

These data were organized manually by the researchers, i.e., without the use of software, and presented in nuclei of meaning, with their respective associated ideas, according to the emerging topics of the data, consolidating the topic “Meanings attributed to voice and insecurities in oral communication from transgender women’s perspective in socialization processes”. It should be added that the analyses were not ratified by the witnesses.

Data interpretation was based on Symbolic Interactionism, which supported the interpretation of the meanings attributed to voice, enabling an understanding of the meanings, actions, and interpretations of transgender women. Symbolic Interactionism analyzes socialization and resocialization, identifying changes in behavior, opinions, perspectives, and social needs that allow individuals to understand and interpret objects, people, and relationships in their environment. The theoretical framework is relevant to the field of health because it involves the development of knowledge, meanings, actions, interpretations, and an understanding of care attitudes and lifestyle practices on a given topic^(16)^.

## RESULTS

### Sociodemographic characterization

The study included 13 trans women, aged 18 to 53, with the following education levels: elementary education (3); high school (5); undergraduate degree (2); specialization (2); and doctoral degree (1). Five participants were not professionally active, but the others reported working as lawyers, makeup artists, hairdressers, artists, journalists, freelancers, and beauticians. Regarding their health conditions, most of them had undergone vocal confirmation surgery and had already seen a speech-language pathologist.

### Meanings attributed to voice and insecurities in oral communication from transgender women’s perspective in socialization processes

It is evident that the meanings attributed to the voice by trans women permeate feelings of fear, shame and acceptance (self and social), in the contexts in which they need to interact and recognize themselves as people in a society with many standards related to gender issues and oppression in the face of singularities and pluralities, with emphasis here on the voice of these trans women.

### “Fear” in the face of self-perception of the voice

Participants perceive their voices as an important element that aligns with gender identification, bringing sensations and feelings that refer to self-acceptance and their identification with themselves and in society, which can be better understood based on the concepts of “mind”, “self” and “society”, introducing a magnifying glass for the comprehensive search.

Furthermore, participants revealed that the vocal adjustments they made in an attempt to feminize their voices can leave them feeling insecure when speaking in public, as they are unable to maintain full control over their vocal emission. The fear that their vocal emission will not match the vocal adjustments previously created to identify the female gender causes feelings of embarrassment, anxiety, and isolation from others, as can be seen in their statements:

*Sometimes we speak and end up sounding lower. And sometimes I avoid speaking because I know people will look at me. No one says anything, no one asks, but I know those looks get left, you know? And that bothers me*. (TW2, 34 years old, self-employed)

*Yesterday, I went out with my friends to a restaurant and my voice was getting mixed up due to the lack of tone variation. Like, when I went to call someone, they wouldn’t hear. I’m afraid to speak louder. I’m afraid my voice will crack.* (TW6, 53 years old, hairdresser)

In the face of fears and efforts, the heteronormative patterns that imply social relations are revealed, which, through participants, reveal the meanings of insecurity and the need for “feminine voices” to meet the search for a “social standard” imposed by society.

*I also like my voice, and I realize that sometimes I make a bit of an effort to keep it more feminine. So, I still have dysphoria, and it still makes me feel insecure.* (TW7, 27, filmmaker)

*I think my voice is pretty neutral, but I wish it were more feminine. Things like laughing at something unexpectedly-sometimes I think it can sound a little more masculine, and it makes me uncomfortable. In a way, I try to accept it, but I feel a bit awkward in that situation.* (TW6, 53, hairdresser)

In fact, the self-perception of voice in everyday life is experienced through fear of orality, with expressions influenced by heteronormativity and the absence of public inclusion in society.

### “Shame” about self-perception of the voice

Participants’ accounts show that the lack of vocal identification begins even before the transgender process. Some characteristics of the vocal sound already caused discomfort even when the person still assumed their biological sex at birth. It is also important to highlight that many people, regardless of gender, report situations of vocal disadvantage and lack of identification with their own voice.

In the case of trans women, it can be seen that gender issues can be evident well before the transition process itself, of which the voice is also a part.

*I never really liked my voice, even before I started my transition. It had been an issue since I was a teenager.* (TW1, 34 years old, self-employed)

[The voice] *It’s something that, for me, is really bad, because even before I transitioned, it was already bad. I already didn’t like it; I’ve always hated my voice, actually.* (TW2, 38 years old, psychologist/lawyer)

Another striking aspect of the study is that self-perception of voice is crucial in the development of these individuals’ vocal identity after transition, as several factors are taken into account in this process of seeking gender identification through voice. Individual personality traits are frequently referenced, and this is associated with the need for a voice that reflects their characteristics.

The voice must represent each person’s “self”, and when it lacks representation, it creates discomfort and distance from themselves and others. Thus, some participants reported feeling ashamed of “forcing” a feminine voice with which they do not identify and becoming “caricatures”.

*I had a dilemma with myself because, while I wanted a feminine voice, I didn’t want to force it. I didn’t want to change my way of speaking too much because my personality, which was already a bit stronger, was more pronounced. So, I didn’t want to force that extremely feminine, “sweet”, “cute” voice, just because it’s considered the most “feminine” voice. So, I didn’t want my voice to become caricatured* [...] *I had a lot of difficulty with this; I was embarrassed to force a more feminine, more nasal voice. That’s why it took me a long time to transition my voice.* (TW3, 32 years old, unemployed)

*I was embarrassed to have a caricatured voice, one that I didn’t recognize myself in. I struggled a lot with this* [...] *and it’s a point I think I’ll always make with every change, because I like my essence. I don’t want anything else, of course not!* (TW4, 25 years old, makeup artist)

In this regard, the process of vocal self-perception is permeated by feelings of shame regarding the intonations and behavioral patterns imposed by society. Previously, feelings of shame accompanied trans women before their gender reassignment process, as they experienced the rites of female vocal standardization and the concerns of the dilemmas of a “caricatured” voice.

### “Acceptance” in the face of self-perception of the voice

A smaller number of participants reported no expectations or discomfort with their voices related to gender expression, demonstrating acceptance of their vocal pattern. This group demonstrates the potential for breaking down social barriers imposed on the trans population, demonstrating that issues other than voice can be highlighted in the process of feminization, such as the body itself and social behavior, which are also important for safe communication without interference in their lives.

*My voice has never limited me in society. I’m a person who likes to move forward, to fight for my place, and to prove myself capable of something. Because my voice is deeper or my voice doesn’t suit my appearance* [...] *I don’t care about that. I don’t care much about other people’s opinions; they won’t make me more insecure, because I’ve always worked on my own acceptance, since I was young. Not the acceptance of others. That’s a human problem. I like myself, I accept myself.* (TW5, 45 years old, beautician)

*For me, I’m 100% satisfied with my voice because I understand that the voice isn’t the main objective or the goal of being feminine. Everything exists, the physical aspect, right? So, I believe there’s no such thing as perfection in anything we do. There’s also the way you act, especially since we have cis women with deep voices.* (TW8, 30 years old, journalist)

*So, I find my voice a bit androgynous, but in a way, it doesn’t bother me as much as other issues I have.* (TW9, 45 years old, professor)

Thus, voice acceptance is experienced by a very small audience, with diverse human subjectivities, and is intrinsic to the patterns that shape the historical and social processes of each participant. These experiences of vocal “acceptance” provide one of the foundations for the diversity and inclusion of trans women.

## DISCUSSION

The process of vocal confirmation and voice use, regardless of gender, is individual, subjective, and representative of socio-historical processes, which leaves the choice of whether or not to undergo vocal changes open. Participants express that fear and shame take on the meaning of having their voice validated and accepted, which is essential for social interaction. On the other hand, when this silences their voice, it increases their vulnerability to social isolation. This echoes the interpretations and actions of caution regarding defense mechanisms in the face of movements and situations that may perpetuate transphobia, as well as being a result of experiencing transphobic circumstances.

It is pointed out that researchers have been using Symbolic Interactionism to verify the ways in which transgender women in eastern Indonesia perceive themselves and their role in society in the face of social stigma and the processes of exclusion they face, building bridges necessary to overcome inequalities^(17)^. Based on the theoretical magnifying glass, it is highlighted that, given the concepts of “mind”, “self”, and “society”, introduced by Blumer^(16)^, the understanding of senses, meanings, and actions is quite diverse. It is based on personal and social issues, showing that several trans women portray satisfaction and dissatisfaction with their voices in multiple contexts, but what prevails is the lack of gender identification through voice.

Hence, strategies to encourage vocal self-perception allow us to observe a person’s level of satisfaction with their own voice and its communicative aspects. Self-perception of vocal disadvantage in trans women is generally related to their daily activities, and the more they use their voice in this routine, the more they feel the impacts of a lack of proper gender identification. Research focusing on voice and communication is needed to develop inclusive healthcare services, especially ensuring that health education encompasses a singular yet plural perspective^(18)^.

Thus, there is a tendency for trans women to seek a voice that matches their gender identity, and this is one of the important issues during the transgenderization process, as it is directly linked to these individuals’ quality of life. A high fundamental frequency (high-pitched voice) is not a defining factor for trans women to have voices appropriate to their gender, as there are inflections specific to the female gender that do not change with an increase in the fundamental frequency of the voice. Therefore, training professionals to provide comprehensive and universal care is essential, especially so that inclusion occurs in services in an equitable and effective manner^(19)^.

The need for inclusive practices in people’s speech is reinforced, as expressive speech is essential, enabling listeners to identify characteristics such as sex, age, social status, and more, primarily through the use of prosody, which encompasses speech intonation, accent patterns, inflections, pauses, and rhythmic variations. People seeking vocal feminization often express concerns about laughter and sneezing, fearing that these acts might be perceived as identifying the male gender^(9,12,13)^.

In this context, national and international knowledge production plays a fundamental role in addressing knowledge gaps, especially regarding the voices of trans women, demonstrating the importance of recognizing trans people’s expressiveness as a facilitating aspect of communicating the gender with which they identify. This contributes to reducing gender dysphoria and improving mental health, social integration, and quality of life^(20-24)^. From this perspective, multidisciplinary training is necessary that recognizes the singularities of trans women based on the social and cultural aspects surrounding vocal care and the transsexualization process^(20)^.

Another important aspect of data production is voice self-assessment or self-perception, which has been highly valued internationally because it attempts to capture a person’s perception of their voice. Since it is a subjective measure, it is widely used for comparison with objective measures taken during speech-language pathology assessments^(18)^. Researchers point out that transgender people’s vocal self-perception has been more negative, demonstrating dissatisfaction with their own voices. Thus, it was seen that the worse an individual’s vocal self-perception, the greater the impact on their quality of life^(19)^.

Self-perception, based on objective and subjective measures of voice quality, is influenced by personality and psychological factors depending on individuals. Personality can influence the perception of vocal therapy success, with individuals with a more positive view of their feminine role demonstrating greater confidence and less concern about how others perceive them, positively influencing the transgenderization process^(17)^. Thus, a voice consistent with gender expression makes communication more expressive in individuals’ socialization situations; otherwise, social interactions generate negative impacts on individuals’ quality of life^(6,9,12,13)^.

A concern regarding the main vocal complaints brought by trans women to voice clinics is the independent development of new vocal adjustments to soften the male-pattern sound and reduce the “embarrassment” of speaking in public and not being recognized as women. These adjustments are highlighted in the literature and can lead to vocal fatigue at the end of the day, as well as a lack of personal identification with the voice used^(25)^.

From a biologically-centered perspective, the structure of the intrinsic laryngeal muscles and the resonators of trans women and transvestites tend to explore lower regions of the vocal pitch, which is socially reflected in the male gender. This causes them social and psychological harm related to gender dysphoria^(26)^.

In an attempt to shape their voices and adapt them to social situations, the emergence of vocal changes among trans people is explained by the effort required in vocal production, as well as by negative adaptations, which are responsible for dysphonia. To minimize this, the search for specialized healthcare services among trans women is increasing, demonstrating the urgency of transforming their vocal patterns for better social integration, given personal and social needs, and improving access to public healthcare services^(7,9,11,18,25)^.

Many trans women experience fear and shame about speaking out, and their voices are perceived as a “social sham”. This is explained by the fact that a deeper voice pattern carries cultural content considered socially conflicting with their physical type, potentially indicating a deceptive female body. Thus, vocal self-image becomes clearer for this group when the therapeutic process and expressive aspects of the voice are presented as gender-identifying factors, but variations in rhythm, melody, inflections, among others, are part of this communicative process^(27)^.

However, the voice should not create any kind of expectation or discomfort regarding gender expression, especially for those who do not seek expressions of femininity or masculinity that fit binary and heteronormative standards. Researchers^(25,27)^ also point out that, among the group of trans women and transvestites, satisfaction with the current condition of their own voice may occur.

Transgender women often seek passability in order to approach the standards conceived as feminine in society. However, many of these individuals show no interest in appearing cisgender and simply seek the comfort of avoiding transphobic situations. It is important to note that the decision regarding oral communication adjustments will always be a shared construct between the individual and the speech-language pathologist, taking into account subjectivity. Therefore, the vocal analysis of transgender women should be conducted according to individuals’ own perspective and life goals^(27)^.

### Study limitations

The limitations of this study include difficulties in accessing this population, reducing the number of participants, the use of focus groups in virtual environments, which may lead to a reduction in researcher-researched interaction and the lack of feedback for participants regarding the results of the study.

### Contributions to nursing, health and public policy

Considering the complexities linked to the quality of healthcare for trans women in Brazil, the study contributes to issues related to the need for an expansion of speech-language pathology services and its interprofessional articulation with other areas of health. Vocal self-perception, as expressed in the statements, points to singularities and common traits, particularly fear, shame, and self-acceptance. These findings highlight situations of social exclusion and vulnerability, and that the general population’s lack of knowledge about gender issues leads to prejudice and insecurity among trans women when trying to coexist in society.

## FINAL CONSIDERATIONS

The study reveals the meaning trans women attributed to their own voices in various spheres, considering personal and social issues that relate to interaction. Vocal self-perception, expressed in the statements, points to singularities and common traits, highlighting fear, shame, and self-acceptance. These findings highlight situations of social exclusion and vulnerability, and that the general population’s lack of knowledge about gender issues leads to prejudice and insecurity among trans women when trying to coexist in society.

Care for the voice of this population must encompass a broader perspective beyond gender issues. Therefore, the individualities and pluralities of this population must be considered from this perspective. Furthermore, the importance of implementing consistent public policies that provide trans people with access to comprehensive physical and mental healthcare is reinforced.

It is important to highlight that, in this study, the interpretive lens of Symbolic Interactionism enabled us to elucidate the meanings attributed to voice by participants, as their perceptions are created and recreated based on the relationships established with themselves (what “I” think of my voice) and with others (how the people with whom “I” interact see me through my voice). This interpretive perspective deepens the understanding and brings to light the concepts of “mind”, “self”, and “society”, enabling the researcher to understand this intertwining of voice with gender issues from the perspective of trans women.

Furthermore, there is a need for future qualitative research based on intersectionality, particularly on aspects of race, culture, social context, and Brazilian regions, given the multidimensional modes of social interaction in Brazil. In the meantime, there is a short-term need for developments regarding interprofessional team care in primary healthcare and its connectivity with specialized care, enabling the overcoming of care barriers to this vulnerable population.

## Data Availability

The research data are available within the article.
