# Supplementary material for: Meanings attributed to the voice by transgender women in socialization processes
Source: Rev Bras Enferm. 2026 Jun 12;79:e20250168. doi: 10.1590/0034-7167-2025-0168 (PMC13262872; doi:10.1590/0034-7167-2025-0168)
Supplement: Translation

# Translation: Sentidos atribuídos à voz por mulheres transgênero nos processos de socialização

**DOI:** 10.1590/0034-7167-2025-0168pt

**Authors:** Tiago José Nunes de Aguiar, Christina César Praça Brasil, Jonas Loiola Gonçalves, Francisca Francisete de Sousa Nunes Queiroz, Waléria Tomaz Pacífico, Juliana Carneiro Melo

**Affiliations:** I Universidade de Fortaleza. Fortaleza, Ceará, Brasil; II Universidade Estadual do Ceará. Fortaleza, Ceará, Brasil

**Keywords:** Mulheres Transgênero, Identidade de Gênero, Voz, Fonoaudiologia, Inclusão Social.

## Abstract

**Objetivos:**

compreender os sentidos atribuídos à voz por mulheres transgênero nos processos de socialização.

**Métodos:**

pesquisa qualitativa, contemplando 13 mulheres transgênero. O estudo foi desenvolvido com grupos focais. Analisaram-se os dados a partir da análise de conteúdo na modalidade temática, interpretando-os à luz do Interacionismo Simbólico.

**Resultados:**

entre as temáticas emergentes, para este recorte de estudo, selecionou-se a seguinte: “Significados atribuídos à voz e inseguranças na comunicação oral na perspectiva de mulheres transgênero nos processos de socialização”, evidenciando que os sentidos atribuídos à voz perpassam sentimentos de medo, vergonha e aceitação (própria e social).

**Conclusões:**

a voz para mulheres transgênero é um recurso essencial para sua inclusão, e aceitação pessoal e social, requerendo atenção do sistema de saúde para favorecer o cuidado integral e reduzir a vulnerabilidade dessa população. Estudos qualitativos evidenciam nuances subjetivas que podem auxiliar na implementação de serviços de saúde interdisciplinares.

## INTRODUÇÃO

As desigualdades de gênero são compreendidas como um fenômeno que envolve processos sócio-históricos e culturais, sendo as relações de poder desiguais e, muitas vezes, atreladas a hierarquias entre o ser “homem” ou “mulher”. Esses processos podem atuar como artefatos para exclusões sociais, “normalidades” e repetidas pedagogias (des)afetivas atribuídas ao público de mulheres^(1)^.

As pessoas transgênero são aquelas que não se identificam com o seu sexo biológico declarado ao nascimento por terceiros, podendo sofrer, com isso, repetidas exclusões sociais identitárias. Em busca da identidade de gênero, a população transgênero (ou população trans) recorre aos serviços de saúde para realizar o processo de transição, na busca da sua construção social e pertencimento. Muitas vezes, isso pode ser motivado pela opressão do movimento da heteronormatividade incorporado à sociedade machista e sexista^(2)^.

No cenário brasileiro, mesmo com a existência do Sistema Único de Saúde (SUS), muitas mulheres trans ainda sofrem com as dificuldades de acesso aos serviços de saúde, principalmente pela quase inexistência de informações sobre uma população ainda estigmatizada e vulnerabilizada^(2-5)^. Nos últimos anos, a procura de mulheres transgênero (ou mulheres trans) por serviços públicos de saúde especializados é crescente, principalmente por aquelas que buscam a cirurgia de redesignação sexual ou transgenitalização (cirurgia para adequação do sexo anatômico ao gênero que a pessoa se reconhece). Ademais, a ausência de cuidado estruturado para a população trans e o preconceito dos profissionais nos serviços de saúde são percebidos como barreiras para reconhecer que a população deve ser atendida em todos os níveis de atenção à saúde. Isso evidencia uma assistência fragilizada tanto nas experiências internacionais quanto nas nacionais^(3-8)^.

Importante destacar que a capacitação da equipe interdisciplinar é um aspecto fundamental no processo de transexualização, pois este envolve uma ampla transformação do corpo, comportamento, concepções sociais e filosóficas, e imagem equivalentes à identidade de gênero; e essas mudanças requerem que o indivíduo seja informado e apoiado nessa trajetória^(9,10)^. Para isso, médicos, psicólogos, fonoaudiólogos e outros profissionais trazem contribuições importantes nesse contexto, uma vez que as adequações na saúde física e mental são uma realidade, perpassando todas as etapas desde o momento em que a pessoa assume a sua identidade de gênero. Assim, a voz, enquanto elemento de expressividade, precisa ser cuidada e observada, facilitando à pessoa trans maior conforto e qualidade de vida no que se relaciona à saúde vocal e às relações sociais^(1,5,11)^.

A voz é o principal meio de comunicação do ser humano, uma vez que, associada à fala, carrega elementos prosódicos, variantes segmentais, padrões rítmicos e ajustes de qualidade vocal, formando a identidade vocal da pessoa^(9-11)^. No contexto das mulheres trans, a voz desempenha um papel fundamental na construção da identidade. Por isso, tem sido apontada como uma das principais queixas desse público, uma vez que a identificação da voz masculina em uma mulher trans pelo ouvinte traz para ela insegurança, medo, vergonha e constrangimentos sociais. Há, inclusive, na literatura e na mídia em geral diversos registros de preconceitos, agressões físicas e verbais contra esse grupo^(12)^.

Assim, muitas delas procuram realizar tratamentos fonoaudiológicos e recorrem também a cirurgias no intuito de serem identificadas no gênero feminino em situações de vida diária, principalmente nos casos em que o ouvinte não dispõe de pistas visuais. Para disfarçar suas vozes no meio social, as mulheres trans acabam por realizar ajustes em seus aparelhos fonadores para caracterizar uma voz feminina. Porém, muitas vezes, essa “nova voz” fica bem distante dos padrões vocais expressos na heteronormatividade imposta pela sociedade^(8,9)^.

Essa inadequação na voz das mulheres trans é ocasionada pela falta de orientação profissional, o que leva ao extremo cansaço vocal ao fim do dia e falta de identificação da voz utilizada. Elas também relatam que esses ajustes vocais são impossíveis de serem mantidos por um longo período e acabam sendo percebidos pelos ouvintes. Ademais, as mulheres trans passam por situações de julgamento de suas vozes diariamente pelos ouvintes, o que pode ter impacto significativo nas suas subjetividades e pluralidades durante a construção da nova identidade de gênero^(12,13)^.

Na realidade brasileira, ocorrem a necessidade da produção do conhecimento acerca da percepção sobre a voz das mulheres trans e o processo de socialização, visto a ausência da produção consolidada em uma perspectiva interacionista. De tal modo, a construção dessas evidências é urgente, diante dos chamamentos da superação das lacunas e melhoria do cuidado em saúde. O conhecimento contribui para construção do cuidado integral e para alcançarmos os objetivos dos Objetivos do Desenvolvimento Sustentável, em especial o objetivo 3, que versa sobre o asseguramento e promoção de uma vida saudável para todas as pessoas.

Assim, é imperativo a ampliação do olhar integral à saúde da mulher transgênero, favorecendo a socialização dessa população, e questiona-se: quais sentidos são atribuídos à voz pelas mulheres trans nos processos de socialização?

## OBJETIVOS

Compreender os sentidos atribuídos à voz por mulheres transgênero nos processos de socialização.

## MÉTODOS

### Aspectos éticos

A presente pesquisa foi aprovada pelo Comitê de Ética em Pesquisa da Universidade de Fortaleza. Destaca-se que, para proteger as identidades das participantes, adotou-se como codificação o uso de letras, números, idade e ocupação, referindo-se à, por exemplo, “MT1, idade e ocupação”, e assim, sucessivamente. Complementa-se que todas as participantes foram convidadas e aceitaram a participação no estudo mediante a leitura e assinatura do Termo de Consentimento Livre e Esclarecido.

### Tipo de estudo

Estudo qualitativo^(14)^, guiado pelo *COnsolidated criteria for REporting Qualitative research* ^(15)^. A abordagem utilizada perpassa a subjetividade das mulheres trans e seus processos sócio-históricos. Aponta-se que, no escopo desta investigação, a análise dos seguintes aspectos é fundamental: cultura, sentimentos, crenças, percepções, hábitos, conhecimentos, experiências de vida das participantes, entre outros^(14)^.

Destarte, a tipologia insere na produção do conhecimento nuances para compreensão dos sentidos da voz pelas mulheres trans nos processos de socialização, visto que, pelos contextos de omissão de cuidados nos serviços de saúde, como também pelas práticas opressoras da sociedade, essas muitas vezes são objetivadas e negligenciadas. Assim, a pesquisa qualitativa introduz perspectiva para escuta qualificada desse público, tendo como base o respeito aos seus processos sociais e suas historicidades^(12,14)^.

### Cenário de pesquisa e seleção das participantes

O estudo foi realizado no período de agosto a dezembro de 2023. Inicialmente, realizou-se a identificação de dois locais de referência no atendimento a pessoas transgênero para execução da pesquisa, ambos localizados na cidade de Fortaleza, Ceará, Brasil. Um dos locais foi uma clínica particular com atuação de profissionais das áreas de otorrinolaringologia e fonoaudiologia que atendem à demanda de pacientes a partir de convênios de saúde e particulares.

Ressalta-se que as pacientes trans atendidas nesse serviço são residentes de Fortaleza, Ceará, outros estados brasileiros e de outros países. Outro local de investigação foi um serviço de saúde vinculado a uma universidade particular privada, onde se realizam atendimentos à população trans pelo SUS e por convênios, com ambulatórios especializados nas áreas de psicologia, fonoaudiologia, otorrinolaringologia e ginecologia.

Na segunda etapa, foram definidas as participantes a partir do método bola de neve, uma estratégia para obtenção que se baseia na utilização de cadeias de referência. A técnica é útil para estudar grupos de acesso restrito ou difícil, no qual possibilitou o recrutamento do público-alvo e, a partir dos critérios de elegibilidade, a entrada no estudo^(14)^. A busca pelas mulheres trans partiu dos dois serviços de saúde mencionados anteriormente, onde foram afixados cartazes físicos e postados convites nas redes socais (*Instagram^®^*). Nesse processo, obtiveram-se as informantes-chave, as quais indicaram outras participantes para a composição do estudo, abordando as mesmas para sua participação no estudo a partir dessas estratégias.

Os critérios de inclusão foram centrados em: pessoas que se identificam como mulher transgênero ou transexual com idade de 18 a 60 anos (pois a fase adulta exclui os aspectos de mudança vocal e o envelhecimento da voz decorrente da idade); mulheres trans brasileiras, podendo residir em qualquer cidade brasileira ou em outros países; e que tivessem vinculação com os serviços sob investigação ou com mulheres atendidas nesses serviços. Assim, foram identificadas 13 mulheres trans aptas a compor o universo do estudo.

### Produção dos dados

A produção dos dados ocorreu em duas etapas. Inicialmente, aplicou-se um questionário socioeconômico e de condições de saúde de mulheres trans, que foi elaborado pelos próprios pesquisadores para obtenção de dados sobre idade, condições de saúde, idade inicial da transição de gênero, profissão, tratamento hormonal, acesso a tratamento fonoaudiológico e otorrinolaringológico, condições vocais atuais, entre outros aspectos.

Em conseguinte, desenvolveu-se um grupo focal, que abrangeu questões norteadoras com temáticas elaboradas a partir das premissas do Interacionismo Simbólico (sentidos, ações e interpretações), abordando os seguintes aspectos: percepções e sentimentos sobre a identificação de gênero por meio da voz; associações entre qualidade de vida e voz; interferências da voz nas relações sociais e de trabalho; dores e angústias que associam a voz ao processo transexualizador; impactos psicossociais percebidos pela condição vocal; esperanças e necessidades das mulheres trans relacionadas à saúde geral e vocal.

Destaca-se que os dois grupos focais foram realizados virtualmente, conduzidos por dois fonoaudiólogos, do gênero masculino e feminino, ambos com experiência prévia em pesquisa qualitativa e treinados com o instrumento do estudo. Assim, definiram-se datas e horários, acordados entre as participantes e os pesquisadores, utilizado uma conta do *Google Meet^®^* com licença *Meet Pro*, com a disponibilização de um *link* para a sessão e, posteriormente, o material produzido baixado para arquivo pessoal dos pesquisadores e complementado com as anotações do diário de campo.

### Organização e análise dos dados

Os dados foram transcritos com base nas gravações e anotações, visando uma maior fidedignidade, riqueza de detalhes e complementações necessárias aos registros. Em seguimento, foram tratados com base na análise de conteúdo na modalidade temática^(14)^, a qual corresponde a um método que engloba um conjunto de estratégias que possibilitam a análise de diversos tipos de comunicação, com o objetivo de obter um maior conhecimento sobre o assunto em pauta dentro de um contexto mais específico.

Esses dados foram organizados de forma manual pelos pesquisadores, ou seja, sem aporte de *software* e apresentados em núcleos de sentido, com as respectivas ideias associadas, de acordo com as temáticas emergentes dos dados, consolidando a temática “Significados atribuídos à voz e inseguranças na comunicação oral na perspectiva de mulheres transgênero nos processos de socialização”. Complementa-se que as análises não foram ratificadas pelas depoentes.

A interpretação dos dados amparou-se no Interacionismo Simbólico, que respaldou a interpretação dos significados atribuídos à voz, possibilitando a compreensão dos sentidos, ações e interpretações das mulheres transgênero. O Interacionismo Simbólico analisa a socialização e a ressocialização, identificando mudanças de comportamento, opiniões, perspectivas e necessidades sociais que permitem ao indivíduo compreender e interpretar objetos, pessoas, e relacionamentos no ambiente. A lupa teórica é relevante para o campo da saúde, por envolver o desenvolvimento de conhecimentos, significados, ações, interpretações, e compreensão de atitudes de cuidado e práticas de estilo de vida sobre determinado tema^(16)^.

## RESULTADOS

### Caracterização sociodemográfica

O estudo contou com a participação de 13 mulheres trans, com idades de 18 a 53 anos e os seguintes níveis de escolaridade: ensino fundamental (3); ensino médio (5); graduação (2); especialização (2); e doutorado (1). Cinco participantes não exercem atividade profissional, porém as demais referiram atuar como advogada, maquiadora, cabeleireira, artista, jornalista, autônoma e esteticista. Em relação às condições de saúde, a maioria delas realizou a cirurgia de confirmação vocal e já teve consultas com o fonoaudiólogo.

### Significados atribuídos à voz e inseguranças na comunicação oral na perspectiva de mulheres transgênero nos processos de socialização

Evidencia-se que os sentidos atribuídos à voz pelas mulheres trans perpassam sentimentos de medo, vergonha e aceitação (própria e social) nos contextos em que precisam interagir e se reconhecer como pessoas em uma sociedade com muitos padrões relacionados às questões de gênero e opressões, diante das singularidades e pluralidades, com ênfase aqui para a voz dessas mulheres trans.

### “Medo” diante da autopercepção da voz

As participantes percebem suas vozes como um importante elemento que se alinha à identificação de gênero, trazendo sensações e sentimentos que remetem à autoaceitação e à identificação delas consigo mesmas e na sociedade, o que pode ser mais bem compreendido com base nos conceitos de “*mind*”, “*self*” e “*society*”, introduzindo como lupa para a busca compreensiva.

Ademais, revela-se entre as participantes que os ajustes vocais por elas elaborados, na busca de feminilizar a voz, podem deixá-las em situações de insegurança ao falar em público, por não conseguirem manter o controle total da emissão vocal. O medo da emissão sonora não corresponder aos ajustes vocais previamente criados para identificação do gênero feminino causa sensações de constrangimento, ansiedade e isolamento diante dos outros, como se pode observar nas falas:

*A gente, às vezes, fala e acaba saindo um tom mais grave. E às vezes, evito falar porque eu sei que as pessoas vão me olhar. Ninguém fala nada, ninguém pergunta, mas eu sei que ficam aqueles olhares, entendeu? E isso me incomoda.* (MT2, 34 anos, autônoma)

*Ontem, eu saí com meus amigos para um restaurante e a minha voz interferiu pela falta de variação de tom. Tipo, quando eu ia chamar alguém, essa pessoa não escutava. Eu tenho medo de falar mais alto. Tenho medo que minha voz falhe.* (MT6, 53 anos, cabeleireira)

Diante dos medos e esforços, revelam-se os padrões heteronormativos que implicam as relações sociais, que, por meio das participantes, revelam os significados de insegurança e a necessidade de “vozes femininas” para atender à busca de um “padrão social” imposto pela sociedade.

*Eu também gosto da minha voz, e eu percebo que, às vezes, eu faço um pouco de esforço para tentar manter ela* [a voz] *mais feminina. Então, eu ainda tenho disforia e ainda é uma coisa que me deixa insegura.* (MT7, 27 anos, cineasta)

*Eu acho minha voz bem neutra, mas eu gostaria que fosse mais feminina. Questões do tipo você rir de alguma coisa de forma inesperada, às vezes eu acho que pode soar um pouco mais masculino e me deixa incomodada. De certa forma, eu tento aceitar isso, só que eu fico meio desajeitada com essa situação.* (MT6, 53 anos, cabeleireira)

Com efeito, a autopercepção da voz no cotidiano é vivenciada pelo medo diante da oralidade, com expressões influenciadas pela heteronormatividade e ausência da inclusão do público na sociedade.

### “Vergonha” diante da autopercepção da voz

Os relatos das participantes evidenciam que a falta de identificação vocal se inicia antes mesmo do processo de transexualização. Algumas características do traço sonoro vocal já geravam incômodos mesmo quando a pessoa ainda assumia o sexo biológico do nascimento. Também é importante destacar que muitas pessoas, independentemente do gênero, referem situações de desvantagem vocal e falta de identificação com a própria voz.

No caso de mulheres trans, pode-se verificar que as questões de gênero podem ser evidenciadas bem antes do processo de transição propriamente dito, do qual a voz também faz parte.

*Eu nunca gostei muito da minha voz até mesmo antes de começar a minha transição. Já era uma questão desde adolescente.* (MT1, 34 anos, autônoma)

[A voz] *É algo que, para mim, é muito ruim, porque, desde quando eu ainda não havia transicionado, já era ruim. Eu já não gostava, sempre detestei a minha voz, na verdade.* (MT2, 38 anos, psicóloga/advogada)

Outro aspecto que chama atenção no estudo é o fato de que a autopercepção da voz é determinante na construção da identidade vocal dessas pessoas após a transição, uma vez que vários fatores são levados em consideração nesse processo de busca pela identificação de gênero através da voz. Os traços de personalidade individuais são muito referidos, e isso se associa à necessidade de a pessoa ter uma voz com as suas características.

A voz tem que representar o “eu” de cada um, e quando esta não tem representatividade, gera desconforto e distanciamento da pessoa de si própria e dos outros. Desta forma, algumas participantes referem ter vergonha de “forçar” uma voz feminina com a qual não se identificam e de ficarem “caricatas”.

*Eu tinha um dilema comigo mesma, porque, ao mesmo tempo que eu queria uma voz feminina, eu não queria forçar. Eu não queria mudar tanto meu jeito de falar porque minha personalidade, que já um pouco mais forte. Então, eu não queria forçar aquela voz extremamente feminina, “meiguinha”, “fofinha”, só porque é a voz considerada mais “feminina”. Então, eu não queria que minha voz ficasse caricata* [...] *eu tive muita dificuldade nisso, eu ficava com vergonha de ficar forçando uma voz mais feminina, mais anasalada. Por isso, demorei muito para transicionar minha voz.* (MT3, 32 anos, sem ocupação)

*Tinha vergonha de ficar com uma voz caricata, que eu não me reconhecesse. Eu bati muito nesse ponto* [...] *e é um ponto que eu acho que eu sempre vou bater em todas as modificações, porque eu gosto da minha essência. Eu não quero outra, claro que não!* (MT4, 25 anos, maquiadora)

Nesse sentido, o processo da autopercepção vocal é permeado pelo sentimento de vergonha diante das entonações e padrões comportamentais impostos pela sociedade. Outrora, o sentimento de vergonha acompanha as mulheres trans antes do seu processo transexualizador, que vivenciam os ritos da padronização vocal feminina e as preocupações dos dilemas da voz “caricata”.

### “Aceitação” diante da autopercepção da voz

Uma quantidade menor de participantes não referiu nenhum tipo de expectativa ou desconforto com a voz relacionada à expressão de gênero, revelando aceitação do seu padrão vocal. Esse grupo mostra as possibilidades de ruptura das barreiras sociais impostas à população trans, evidenciando que outras questões além da voz podem ser destacadas no processo de feminilização, a exemplo do próprio corpo e das maneiras de agir socialmente, que são também importantes para uma comunicação segura e sem interferências em suas vidas.

*A voz nunca me limitou na sociedade. Sou uma pessoa que gosta de seguir em frente, de lutar pelo meu espaço e de me mostrar capaz de algo, porque a minha voz é mais grave ou a minha voz não é adequada à minha aparência* [...] *eu não quero saber disso. Não me importa muito a opinião alheia, não vai me deixar mais insegura, porque eu sempre trabalhei na minha cabeça, desde jovem, a minha própria aceitação. Não a aceitação alheia. Isso é um problema das pessoas. Eu gosto de mim, eu me aceito.* (MT5, 45 anos, esteticista)

*Para mim, eu estou 100% satisfeita com a minha voz, porque eu tenho uma concepção de que a voz não é o objeto principal ou a meta de ser feminina. Existe tudo, o corporal, né? Então, acredito que não existe a perfeição em nada do que nós vamos fazer. Também existe a maneira de como você age, até porque temos mulheres cis com as vozes graves.* (MT8, 30 anos, jornalista)

*Então, eu acho a minha voz meio andrógena, mas, de certa forma, não me incomoda tanto quanto outras questões que eu tenho.* (MT9, 45 anos, professora universitária)

De tal modo, a aceitação da voz é vivenciada em um público muito pequeno, com variadas subjetividades humanas, sendo intrínseca aos padrões que moldam os processos históricos e sociais de cada participante. Essas vivências da “aceitação” vocal inserem um dos alicerces para a diversidade e inclusão das mulheres trans.

## DISCUSSÃO

O processo de confirmação vocal e uso da voz, independentemente do gênero, é individual, subjetivo e representativo pelos processos sócio-históricos, o que deixa livre a escolha por mudanças vocais ou não. As participantes manifestam que o medo e a vergonha assumem o sentido de ter a voz validada e aceita, o que é essencial para interação social. Por outro lado, quando essa silencia sua voz, aumenta sua vulnerabilidade para isolamento social. Isso ecoa com as interpretações e ações com a cautela para os mecanismos de defesa diante de movimentos e situações que podem perpetuar a transfobia, como também ser um resultado da vivência de circunstâncias transfóbicas.

Aponta-se que pesquisadores vêm utilizando o Interacionismo Simbólico para verificar as formas como mulheres transexuais da região leste da Indonésia percebem a si mesmas e seu papel na sociedade diante do estigma social e dos processos de exclusão que enfrentam, construindo pontes necessárias para a superação das desigualdades^(17)^. Com base na lupa teórica, destaca-se que, diante dos conceitos de “*mind*”, “*self*” e “*society*”, trazidos por Blumer^(16)^, a compreensão de sentidos, significados e ações é bastante diversa. Baseia-se em questões pessoais e sociais, mostrando que várias mulheres trans retratam satisfações e insatisfações com suas vozes em múltiplos contextos, porém o que prevalece é a falta de identificação de gênero por meio da voz.

Nesse sentido, as estratégias de estímulo à autopercepção vocal possibilitam observar o grau de satisfação da pessoa com a sua própria voz e seus aspectos comunicativos. A autopercepção de desvantagem vocal em mulheres trans, geralmente, está relacionada às suas atividades de vida diária, e quanto mais uso da voz elas fazem nessa rotina, mais sentem os impactos da falta de identificação adequada do gênero. Insere-se a necessidade das pesquisas com foco em voz e comunicação, objetivando a construção de serviços de saúde inclusivos, principalmente que a formação na saúde perpasse um olhar singular e ao mesmo tempo plural^(18)^.

Destarte, existe uma tendência pela busca da voz correspondente à identidade de gênero na mulher trans, e isso é uma das questões importantes durante o processo transexualizador, pois está diretamente ligada à qualidade de vida dessas pessoas. A frequência fundamental elevada (voz aguda) não é algo definidor para que mulheres trans tenham suas vozes adequadas ao gênero a que pertencem, pois existem inflexões específicas do gênero feminino que não se alteram com o aumento da frequência fundamental da voz. Nesse sentido, é essencial a formação de profissionais para produção do cuidado de forma integral e universal, principalmente para que a inclusão ocorra nos serviços de forma equânime e resolutiva^(19)^.

Reforça-se a necessidade de práticas inclusivas diante do discurso das pessoas, pois a expressividade de fala se faz necessária, sendo capaz de identificar no ouvinte características como sexo, idade, condição social, entre outros, principalmente pelo uso da prosódia, que abrange as entonações de fala, padrões de acento, inflexões, pausas e variações rítmicas. As pessoas que buscam a feminilização vocal, muitas vezes, exibem preocupações em situações de risadas e espirros, por medo desses atos soarem como identificador do gênero masculino^(9,12,13)^.

Nesse contexto, a produção do conhecimento em caráter nacional e internacional desempenha papel fundamental na busca de compreender lacunas do conhecimento, em especial sobre a voz de mulheres trans, mostrando a importância em reconhecer a expressividade da pessoa trans como um aspecto facilitador da comunicação do gênero com o qual a pessoa se identifica. Isto colabora com a redução da disforia de gênero, na melhora da saúde mental, integração social e qualidade de vida^(20-24)^. Nessa perspectiva, é necessária uma formação multiprofissional que reconheça as singularidades das mulheres trans com base nos aspectos sociais e culturais que circundam o cuidado vocal e o processo transexualizador^(20)^.

Outro ponto importante a partir da produção dos dados é a autoavaliação ou autopercepção da voz, que tem sido altamente valorizada internacionalmente, pois tenta capturar a percepção que a pessoa tem da sua voz. Por ser uma medida subjetiva, é amplamente utilizada para comparação com medidas objetivas tomadas durante o processo de avaliação fonoaudiológica^(18)^. Pesquisadores apontam que a autopercepção vocal de pessoas transexuais tem sido mais negativa, demonstrando insatisfação com suas próprias vozes. Desta forma, viu-se que, quanto pior a autopercepção vocal do indivíduo, maior é o impacto na sua qualidade de vida^(19)^.

A autopercepção, com base em medidas objetivas e subjetivas da qualidade da voz, sofre influência da personalidade e de fatores psicológicos de acordo com cada indivíduo. A personalidade pode influenciar a percepção do sucesso terapêutico vocal, em que indivíduos com uma visão mais positiva em relação ao seu papel feminino demonstram mais confiança e menos preocupações de como os outros os veem, influenciado positivamente no processo de transexualização^(17)^. Assim, a voz condizente com a expressão de gênero deixa mais expressiva a comunicação nas situações de socialização do indivíduo; caso contrário, as interações sociais gerar impactos negativos na qualidade de vida do sujeito^(6,9,12,13)^.

Uma preocupação diante das principais queixas vocais trazidas por mulheres trans aos ambulatórios de voz é a criação, de forma independente, de novos ajustes vocais para amenizar o traço sonoro do padrão masculino e reduzir a “vergonha” de falar em público e não serem reconhecidas como mulheres. Esses ajustes são destacados na literatura e podem levar à fadiga vocal ao final do dia, como também falta de identificação pessoal com a voz utilizada^(25)^.

Do ponto de vista biológico-centrado, a estrutura dos músculos intrínsecos da laringe e os ressoadores de mulheres trans e travestis tendem a explorar regiões graves do *pitch* vocal, sendo refletido socialmente ao gênero masculino. Isto lhes traz prejuízos sociais e psicológicos relacionados à disforia de gênero^(26)^.

Na tentativa de modelar suas vozes e adequá-las socialmente, o surgimento de alterações vocais no grupo de pessoas trans é justificado pelo esforço na produção vocal, assim como nas adaptações negativas, sendo estas responsáveis por quadros de disfonia. Para minimizar isso, a busca pelo acompanhamento por serviços de saúde especializados é crescente no público de mulheres trans, demonstrando urgência na transformação do padrão vocal para sua melhor inserção social, diante das necessidades pessoais e sociais, e melhorias no acesso aos serviços de saúde públicos^(7,9,11,18,25)^.

Muitas mulheres trans vivenciam o temor e a vergonha de falarem, e de suas vozes serem percebidas como uma “farsa social”. Isso se explica pelo fato de o padrão de voz mais grave carregar conteúdo cultural considerado socialmente conflitante com o seu tipo físico, podendo ser denunciante de um corpo feminino enganador. Assim, verifica-se que a autoimagem vocal fica mais clara para este grupo quando o processo terapêutico e os aspectos expressivos da voz são apresentados como fatores identificadores de gênero, mas que variações de ritmo, melodia, inflexões, entre outros, fazem parte desse processo comunicativo^(27)^.

Porém, a voz não pode criar nenhum tipo de expectativa ou desconforto no que diz respeito à expressão de gênero, especialmente para aquelas pessoas que não procuram expressões de feminilidade ou masculinidade que se ajustem aos padrões binários e heteronormativos. Pesquisadores^(25,27)^ apontam ainda que, entre o grupo de mulheres trans e travestis, pode ocorrer a satisfação com a condição atual da própria voz.

A passabilidade, muitas vezes buscada por mulheres trans, vem no sentido de se aproximar dos padrões concebidos como feminino em sociedade, porém várias dessas pessoas não demonstram interesse em parecer uma mulher cisgênero e buscam apenas ter o conforto de não passar por situações de transfobia. Destaca-se que a decisão sobre os ajustes de comunicação oral sempre será uma construção compartilhada entre a pessoa e o fonoaudiólogo considerando a subjetividade. Assim, a análise vocal da mulher transgênero deve ser realizada de acordo com a própria perspectiva da pessoa e seus objetivos de vida^(27)^.

### Limitações do estudo

As limitações do estudo perpassam as dificuldades de acesso a essa população, reduzindo o universo de participantes e a utilização de grupos focais em ambiente virtuais, podendo ocorrer a redução da interação pesquisador-pesquisado e a ausência de *feedback* para as participantes quanto aos resultados do estudo.

### Contribuições para a área da enfermagem, saúde e políticas públicas

Considerando as complexidades ligadas à qualidade da assistência em saúde das mulheres trans no Brasil, o estudo contribui com as questões relacionadas à necessidade de uma ampliação do atendimento fonoaudiológico e sua articulação interprofissional com as demais áreas da saúde. A inserção de evidências traz as subjetividades dessa população como também uma apropriação de conhecimentos que perpassa todas as profissões. Os achados mostram a necessidade de cuidado interprofissional e igualitário, tendo como base a promoção da qualidade de vida e a redução das desigualdades diante do acesso aos serviços de saúde no Brasil.

## CONSIDERAÇÕES FINAIS

O estudo mostra o significado que as mulheres trans atribuíram às suas próprias vozes em diversas esferas, considerando questões pessoais e sociais que remetem à interação. A autopercepção vocal, explicitada nas falas, remete a singularidades e traços comuns, destacando-se o medo, a vergonha e a autoaceitação. Esses achados evidenciam situações de exclusão social e vulnerabilidade, e que a falta de conhecimento da população em geral sobre as questões de gênero leva a preconceitos e inseguranças das mulheres trans quando tentam conviver em sociedade.

O cuidado com a voz dessa população deve ter um olhar ampliado para além das questões de gênero. Por isso, devem-se considerar, nessa perspectiva, as individualidades e pluralidades dessa população. Ademais, reforça-se a importância de implementação de políticas públicas consistentes que possam dar acesso às pessoas trans a uma atenção integral à saúde física e mental.

Importante destacar que, neste estudo, a lupa interpretativa do Interacionismo Simbólico possibilitou elucidar os sentidos (significados) atribuídos à voz pelas participantes, pois as percepções delas são criadas e recriadas a partir das relações estabelecidas consigo mesmas (o que “eu” acho da minha voz) e com os outros (como as pessoas com as quais “eu” interajo me veem a partir da minha voz). Essa perspectiva interpretativa aprofunda o olhar e traz os conceitos de “*mind*”, “*self*” e “*society*” à tona, fazendo o investigador compreender esse entrelaçamento da voz com as questões de gênero na perspectiva de mulheres trans.

Ademais, ecoa-se a necessidade de pesquisas qualitativas futuras com base na interseccionalidade, principalmente nos aspectos da raça, cultura, social e regiões brasileiras, diante dos modos de interação social multidimensional no Brasil. No oportuno, é efêmera a necessidade de desdobramentos acerca do cuidado da equipe interprofissional na Atenção Primária à Saúde e sua conectividade com atenção especializada, oportunizando a superação das barreiras assistenciais no cuidado a essa população vulnerabilizada.

## Data Availability

Os dados de pesquisa estão disponíveis no corpo do artigo.
